# Cholesterol Regulates Exosome Release in Cultured Astrocytes

**DOI:** 10.3389/fimmu.2021.722581

**Published:** 2021-10-14

**Authors:** Mohammad Abdullah, Tomohisa Nakamura, Taslima Ferdous, Yuan Gao, Yuxin Chen, Kun Zou, Makoto Michikawa

**Affiliations:** Department of Biochemistry, Nagoya City University Graduate School of Medical Sciences, Nagoya, Japan

**Keywords:** apolipoprotein E, astrocytes, cholesterol, exosome, PI3K, Akt

## Abstract

Exosomes are vesicles secreted by various kinds of cells, and they are rich in cholesterol, sphingomyelin (SM), phosphatidylcholine, and phosphatidylserine. Although cellular sphingolipid-mediated exosome release has been reported, the involvement of other lipid components of cell membranes in the regulation of exosome release is poorly understood. Here, we show that the level of exosome release into conditioned media is significantly reduced in cultured astrocytes prepared from apolipoprotein E (ApoE) knock-out mice when compared to those prepared from wild-type (WT) mice. The reduced level of exosome release was accompanied by elevated levels of cellular cholesterol. The addition of cholesterol to WT astrocytes significantly increased the cellular cholesterol levels and reduced exosome release. PI3K/Akt phosphorylation was enhanced in ApoE-deficient and cholesterol-treated WT astrocytes. In contrast, the depletion of cholesterol in ApoE-deficient astrocytes due to treatment with β-cyclodextrin recovered the exosome release level to a level similar to that in WT astrocytes. In addition, the reduced levels of exosome release due to the addition of cholesterol recovered to the control levels after treatment with a PI3K inhibitor (LY294002). The cholesterol-dependent regulation of exosome release was also confirmed by *in vivo* experiments; that is, exosome levels were significantly reduced in the CSF and blood serum of WT mice that were fed a high-fat diet and had increased cholesterol levels when compared to those in WT mice that were fed a normal diet. These results suggest that exosome release is regulated by cellular cholesterol *via* stimulation of the PI3K/Akt signal pathway.

## Introduction

The fusion of multivesicular bodies with the plasma membrane causes the release of intraluminal vesicles within the multivesicular bodies into the extracellular milieu as exosomes (1, 2). These membrane-bound macrovesicles measure 30 to 100 nm in diameter (3), and they contain proteins (4), lipids (5–7), mRNAs, and microRNAs (8–10). Exosomes have been isolated and purified from biological fluids, such as urine (4), blood (11, 12), and breast milk (13), and from *in vitro* cultures of numerous cell types (5, 6, 14). Their presence and stability in biological fluids, as well as their particular composition, make exosomes a potential source of biomarkers for several diseases (12, 15).

Recent studies have shown the involvement of several proteins and lipids in the biogenesis and release of exosomes. For example, it has been shown that endosomal sorting complexes required for transport proteins (16, 17) and lipid-related proteins, such as phospholipid- and phosphoinositide-specific phospholipase C (18), play a role in exosome secretion. It is reasonable to assume that lipids in the cell membrane may affect exosome release since exosomes are released from cells after the fusion of multivesicular bodies with the plasma membrane, and they are rich in cholesterol, sphingomyelin (SM), phosphatidylcholine, and phosphatidylserine when compared to the donor cells (7). From this point of view, studies have demonstrated that exosome release is mediated by cellular lipids, such as SM and ceramide (19, 20). However, the function of other lipids, such as cholesterol, one of the major lipid components of the cellular membrane, in the regulation of exosome release remains unknown.

Regarding lipid transport in the brain, apolipoprotein E (ApoE) has been shown to be involved in cholesterol transport (21, 22). In the central nervous system, ApoE is one of the major lipid acceptors (23), and it interacts with ATP-binding cassette A1 (24) to remove cholesterol from cells to generate high-density lipoprotein particles (25) in an ApoE-isoform-specific manner (22, 26, 27). Altered ApoE isoform-specific high-density lipoprotein formation and its supply to neural cells *via* ApoE receptors may be causes of the altered cholesterol metabolism in the Alzheimer’s disease (AD) brain. In line with this notion, it is known that the ApoE-knock-out (KO) mouse is a model for atherosclerosis. These lines of evidence led us to perform a study to determine the effect of cholesterol and its transporter, ApoE, on exosome release in cultured astrocytes and *in vivo*.

## Materials and Methods

### Animals

WT C57BL/6J mice were obtained from SLC, Inc. (Shizuoka, Japan). ApoE3-KO C57BL/6J mice were purchased from Charles River Laboratories Japan, Inc. (Yokohama, Japan). All mice were bred on a 12-h light-dark schedule with *ad libitum* access to standard chow (CE-2, CLEA, Shizuoka, Japan) and tap water.

### Cell Culture

Primary cultures of mixed glial cells were prepared from the brain of C57BL/6 mouse pups on postnatal day 1 as described previously (14, 22, 26). Briefly, isolated cortices of the brain were minced, and the cortical fragments were incubated in 0.25% trypsin and 20 μg/ml DNase I in phosphate-buffered saline (8.1 mM Na2HPO_4_, 1.5 mM KH2PO_4_, 137 mM NaCl, and 2.7 mM KCl, pH 7.4) at 37°C for 15 min. The fragments were then dissociated by pipetting to produce single-cell suspensions. The dissociated cells were seeded into a 75-cm^2^ flask at a cell density of 1 × 10^7^ in Dulbecco’s Modified Eagle Medium (DMEM) containing 10% fetal bovine serum, at which point microglia were removed by shaking. After 10 days of incubation *in vitro*, astrocytes in the monolayer were trypsinized (0.1%) and reseeded onto 6-cm^2^ dishes. The astrocyte-rich cultures were maintained in DMEM containing 10% fetal bovine serum until use.

### Cholesterol Depletion and Drug Treatments

A stock solution of methyl-β-CD (Sigma Aldrich, St. Louis, MO, USA) was prepared by dissolving β-CD in MilliQ water to a concentration of 16.5 mM. For cholesterol depletion, astrocytes were treated with β-CD at a final concentration of 0.1, 1, or 2 mM for 24 h at 37°C. Cholesterol (Wako, Osaka, Japan) was dissolved in 100% ethanol to prepare stock solutions with a concentration of 10 mg/ml; these solutions were subsequently diluted and used at a final concentration of 5 or 10 μM. LY294002 (Calbiochem, Tokyo, Japan), a PI3K inhibitor, was dissolved in dimethyl sulfoxide to prepare stock solutions with a concentration of 10 mM; these solutions were subsequently diluted and used at a final concentration of 10 or 20 μM. For each experiment, the culture medium was replaced with DMEM containing the indicated amount of drug without serum, and incubated for the indicated time.

### Western Blot Analysis

The CM were collected, and cells were washed in cold phosphate-buffered saline two times, then collected with a scraper in 100 μl of radioimmunoprecipitation assay buffer containing a cocktail of protease inhibitors purchased from Roche Diagnostics (Indianapolis, IN, USA) and phosphatase inhibitors (Wako, Tokyo, Japan). The cell lysates were transferred to a 1.5-ml microtube and homogenized on ice with a glass homogenizer. The resultant homogenate was centrifuged at 12,000 × rpm for 5 min at 4°C to separate the solution from the pellet fraction. The protein concentrations in the supernatants were determined by a BCA protein assay kit (Pierce, Rockford, IL, USA). An equal volume of CM and equal amount of homogenate protein were mixed with the sampling buffer (100 mM Tris-HCl (pH 7.4), 10% glycerol, 4% sodium dodecyl sulfate, 10% mercaptoethanol, and 0.01% bromophenol blue), and analyzed by 12.5% Tris/glycine sodium dodecyl sulfate-polyacrylamide gel electrophoresis. The separated proteins were electrophoretically transferred onto polyvinylidene difluoride membranes, Immobilon-P (Merck-Millipore, Carrigtwohill, IRL), using a transfer buffer (0.1 M Tris, 0.192 M glycine, and 10% methanol). Membranes were then incubated in a blocking solution consisting of 5% powdered milk in Tris-buffered saline with Tween 20 (10 mmol/L Tris-HCl (pH 8.0), 150 mmol/L NaCl, and 0.1% Tween 20) for 1 h at room temperature. These membranes were then incubated with the respective antibodies at 4°C overnight. The primary antibodies used were rabbit anti-flotillin 1 polyclonal antibody (Sigma Aldrich), mouse anti-HSP90 monoclonal antibody (BD Bioscience, Franklin Lakes, NJ, USA), rabbit anti-ApoE polyclonal antibody (EMD Millipore, Billerica, MA, USA), rabbit anti-Akt polyclonal antibody (Cell Signaling, Beverly, MA, USA), rabbit anti-phospho-Akt polyclonal antibody (Cell Signaling), rabbit anti-PI3K monoclonal antibody (Cell Signaling), rabbit anti-phospho-PI3K polyclonal antibody (Cell Signaling), and mouse anti-α-tubulin monoclonal antibody (Sigma Aldrich). The membranes were washed, then incubated with the appropriate secondary antibody conjugated to horseradish peroxidase. Immunoreactive bands were visualized with ImmunoStar Zeta or ImmunoStar LD (Wako, Osaka, Japan), and analyzed with the Amersham Imager 680 (GE Healthcare Life Science, Marlborough, MA, USA). Signal intensities were quantified by ImageJ (1.46r; Java 1.6.0-20 [64 bit] (NIH, Bethesda, MD, USA).

### ELISA for the Determination of the Exosome Levels

Serum exosome levels were determined by a CD9/CD63 exosome ELISA kit (Cosmo Bio., Tokyo, Japan), which is a sandwich ELISA kit that uses anti-CD63 antibody as a capture antibody and anti-CD9 antibody as a detection antibody, according to the manufacturer’s instructions.

### Determination of the Cholesterol Levels

For the analysis of the cellular cholesterol levels, 1 ml of each lysate solution was transferred into separate clean glass tubes containing 4.0 ml of chloroform/methanol (2:1 v/v). The organic phase was separated from the aqueous phase by centrifugation at 3,000 rpm and 4°C for 15 min, then the cholesterol-containing phase was removed from the bottom to another tube and dried under N_2_ gas. The dried cholesterol was then dissolved in 300 μl of isopropyl alcohol, and each sample was transferred onto 96-well polypropylene plates (Corning Gorilla Glass, Corning, NY, USA) and dried under air flow. The dried content of cholesterol was determined by the Amplex^®^Red Cholesterol Assay Kit (Life Technologies, Eugene, OR, USA).

### Collection of CSF, Blood Serum, and Brain Specimens From Mice

Three-month-old WT mice were fed a high-fat diet or a control diet for four months. All mice were housed under a 12-h dark/light cycle, and had access to the high-fat diet or control diet and water *ad libitum*. At the age of 7 months, the mice were sacrificed by anesthesia. Three mixtures of combination anesthetics were prepared with 0.3 mg/kg of medetomidine (Medetomin, Meiji Seika Pharma Co., Ltd., Tokyo, Japan), 4.0 mg/kg of midazolam (Dormicum, Maruishi Pharmaceutical Co., Ltd., Osaka, Japan), and 5.0 mg/kg of butorphanol (Vetorphale, Meiji Seika Pharma Co., Ltd.), and the volume was adjusted with sterilized saline. The anesthetics were administered to mice by intraperitoneal injection at a volume of 0.01 ml/g of body weight. For the investigations of the extracellular exosome and cholesterol levels, CSF from the cisterna magna, blood serum, and brain were collected from the mice.

### Statistical Analysis

Immunopositive bands that were visualized by western blot analysis were quantified by image analysis software (ImageJ 1.46r; Java 1.6.0-20 [64 bit]). Results are expressed as the mean ± standard error (SE) of three independent experiments. Statistical analysis was performed using one-way analysis of variance (ANOVA) followed by the Bonferroni-Dunn test and Student’s *t*-test.

## Results

**Figure 1:**
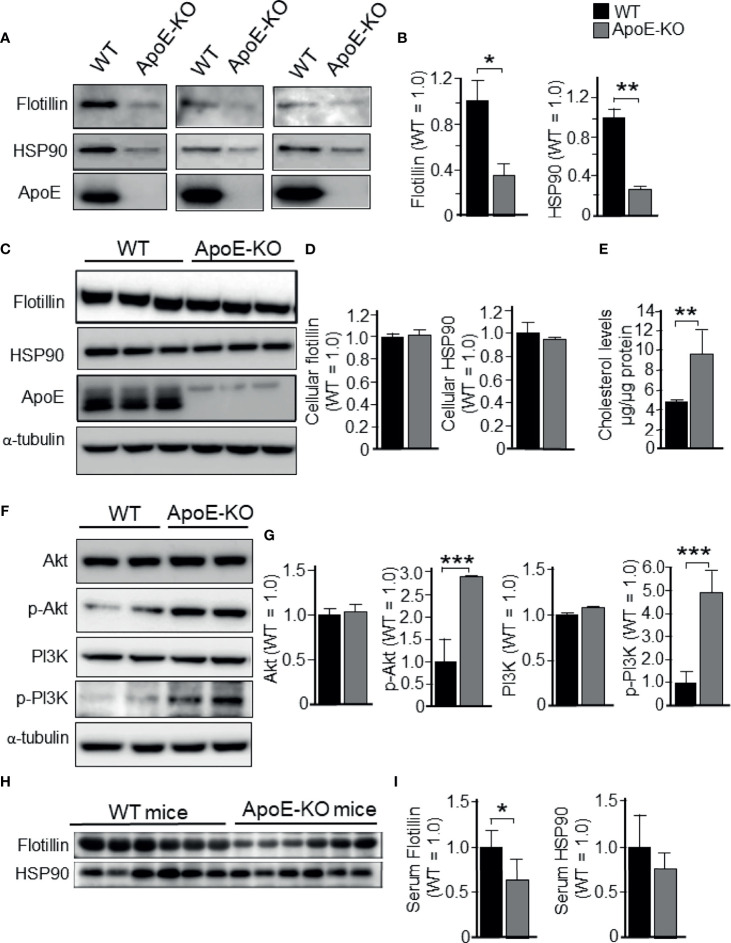
Levels of exosome release in cultured astrocytes prepared from WT and ApoE-KO mouse brain. Astrocyte-rich cultures were prepared from mouse brains as described in the Experimental Procedures section. CM and cell lysates were harvested and subjected to western blot analysis using antibodies against flotillin, HSP90, p-PI3K, pan-PI3K, p-Akt, pan-Akt, and ApoE. WT and ApoE-deficient astrocytes were cultured for 48 h, then the CM **(A, B)** and cell lysates **(C, D)** were obtained and analyzed by western blot analysis. **(E)** The cellular cholesterol levels were determined by a cholesterol determination kit. **(F, G)** Cellular levels of p-PI3K, pan-PI3K, p-Akt, and pan-Akt were determined by western blot analysis using specific antibodies against these molecules. **(H, I)** Flotillin and HSP90 levels in the serum collected from WT and ApoE-KO mice were subjected to western blot analysis using anti-flotillin and HSP90 antibodies. The intensity of each band was quantified by densitometry. Data are expressed as the mean ± SE. n = 3 each. *p < 0.05, **p < 0.01, ***p < 0.001 by Student’s *t*-test.

Firstly, to determine the effect of ApoE deficiency on exosome release from cultured astrocytes into serum-free media, we prepared astrocyte cultures from wild-type (WT) and ApoE-KO mice. Western blot analysis was performed using conditioned media (CM) collected from astrocytes that were cultured for 48 h in serum-free media. When flotillin and heat shock protein 90 (HSP90) were used as exosome markers, we found that exosome release in the CM was significantly reduced in ApoE-deficient astrocytes when compared to WT astrocytes (**Figures 1A, B**). However, the cellular levels of these proteins remained unchanged (**Figures 1C, D**). The cellular cholesterol levels in ApoE-deficient astrocytes were significantly increased when compared to those in WT astrocytes (**Figure 1E**). Since it has been shown that cellular cholesterol regulates exosome release by modulating PI3K and Akt activity (28, 29), we examined the phosphorylation levels of PI3K and Akt. The results showed that the phosphorylation levels of PI3K and Akt were significantly increased in ApoE-deficient astrocytes when compared to WT astrocytes, whereas the total levels of these proteins remained unchanged between ApoE-deficient and WT astrocytes (**Figures 1F, G**). We examined this is also the case for *in vivo*. The serum collected from WT and ApoE-KO mice were subjected to western blot analysis using anti-flotillin and HSP90 antibodies. The levels of flotillin in the serum from ApoE-KO mice was significantly lower than those in the serum from WT mice. The levels of HSP90 shows lower tendency in the serum from ApoE-KO mice compared with those from WT mice (**Figures 1H, I**).

**Figure 2:**
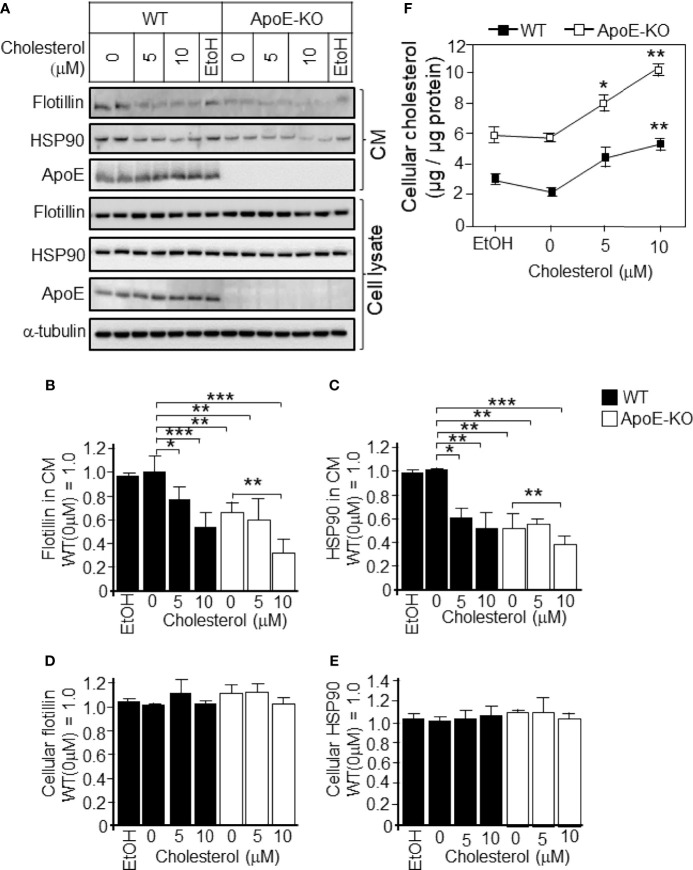
Levels of exosome release in cultured WT and ApoE-deficient astrocytes treated with varying concentrations of cholesterol. Primary astrocyte cultures prepared from WT and ApoE-KO mouse brain were treated with cholesterol at the concentration of 0, 5, or 10 μM. The cultures were then incubated for 48 h, and the CM and cell lysates were harvested. The samples were subjected to western blot analysis using antibodies against flotillin, HSP90, ApoE, and α-tubulin. **(A)** Western blot analysis of the CM and cell lysates. **(B–E)** The signal intensities of the western blots were quantified. **(F)** The cellular cholesterol levels were determined by a cholesterol determination kit. Data are expressed as the mean ± SE. n = 3. *p < 0.05, **p < 0.01, ***p < 0.001 by one-way ANOVA followed by the Bonferroni-Dunn test *versus* EtOH.

To determine whether these changes were caused by cholesterol, we further analyzed the cholesterol-dependent regulation of exosome secretion from cultured astrocytes. Astrocyte cultures prepared from ApoE-KO and WT mice were treated with cholesterol at a concentration of 0, 5, or 10 μM. In WT astrocytes, treatment with cholesterol at the concentrations of 5 and 10 μM significantly reduced the secreted levels of flotillin and HSP90 to levels similar to those of ApoE-deficient astrocyte without cholesterol treatment (**Figures 2A–C**), and there were no changes in the cellular levels of flotillin and HSP90 (**Figures 2A, D, E**). When the WT astrocytes were treated with 5 or 10 μM cholesterol, the cellular cholesterol levels increased to levels similar to those of ApoE-deficient astrocytes without cholesterol treatment (**Figure 2F**). In WT astrocytes, along with the increase in cellular cholesterol levels, the levels of exosome release, as determined by the flotillin and HSP90 levels, were reduced to levels similar to those of ApoE-deficient astrocytes without cholesterol treatment (**Figures 2B, C, F**). Interestingly, levels of ApoE released from WT astrocytes remained unchanged when the cultures were tread with cholesterol, suggesting that cellular cholesterol has no effect on generating ApoE-containing particles (**Figure 2A**).

**Figure 3:**
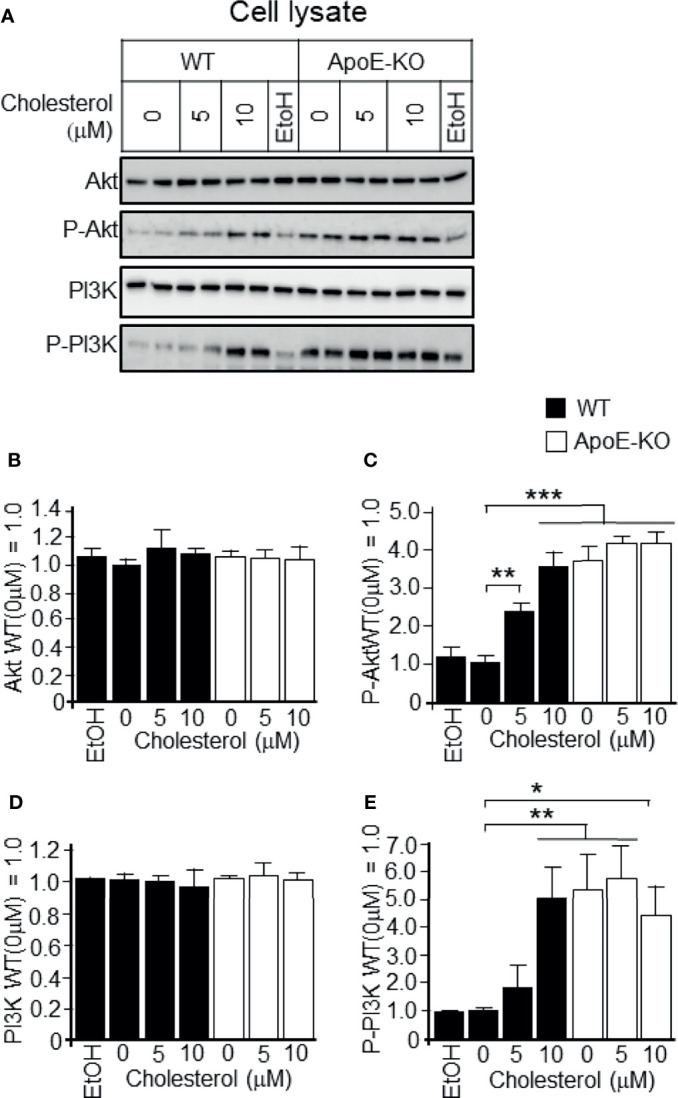
Levels of p-Akt, pan-AKT, p-PI3K, and pan-PI3K in WT and ApoE-deficient astrocytes treated with varying concentrations of cholesterol. Primary astrocyte cultures prepared from WT and ApoE-KO mouse brain were treated with cholesterol at the concentration of 0, 5, or 10 μM. The cultures were then incubated for 48 h, and the cell lysates were harvested. **(A)** The cell lysates were subjected to western blot analysis using antibodies against Akt, p-Akt, PI3K, and p-PI3K. **(B–E)** The intensity of each band was quantified by image analysis software (ImageJ 1.46r; Java 1.6.0-20 [64 bit]). Data are expressed as the mean ± SE. n = 3. *p < 0.05, **p < 0.01, ***p < 0.001 by one-way ANOVA followed by the Bonferroni-Dunn test.

We next determined whether a higher cholesterol level in cells is responsible for the activation of PI3K/Akt. We found that the addition of cholesterol at concentrations of 5 and 10 μM to WT astrocyte cultures significantly increased the levels of phosphorylated PI3K/Akt when compared to WT astrocyte cultures without the addition of cholesterol (**Figures 3A, C, E**). However, cholesterol treatment had no effect on the levels of phosphorylated PI3K/Akt in ApoE-deficient astrocytes (**Figures 3A, C, E**). With respect to the total PI3K/Akt (pan-PI3K/Akt), treatment with cholesterol had no effect in ApoE-deficient astrocytes or WT astrocytes (**Figures 3B, D**).

**Figure 4:**
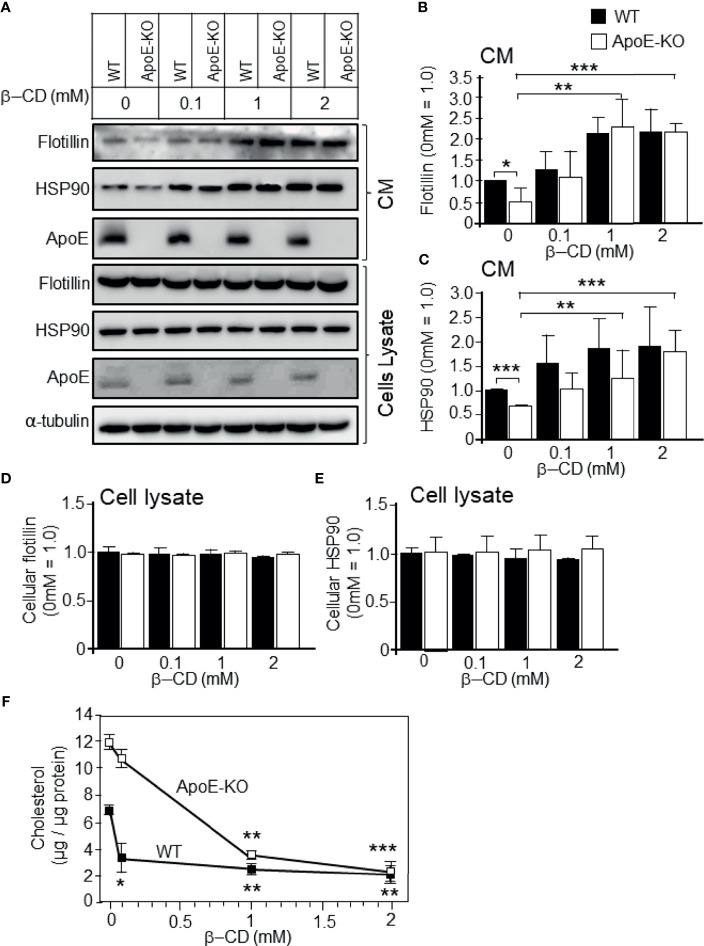
Effect of cholesterol depletion due to β-CD on exosome release. Primary astrocyte cultures prepared from WT and ApoE-KO mouse brain were treated with β-CD at the concentration of 0.1, 1, or 2 mM, and incubated for 24 h. Then, the CM and cell lysates were harvested and subjected to western blot analysis to determine the flotillin, HSP90, and ApoE levels in the CM **(A–C)** and cell lysates **(A, D, E)**. **(B–E)** The intensity of each band was quantified by image analysis software (ImageJ 1.46r; Java 1.6.0-20 [64 bit]). Data are expressed as the mean ± SE. n = 3 each. *p < 0.05, **p < 0.01, ***p < 0.001 by one-way ANOVA followed by the Bonferroni-Dunn test. **(F)** The cellular cholesterol levels were determined by a cholesterol determination kit. Data are expressed as the mean ± SE. n = 3 each. *p < 0.05, **p < 0.01, ***p < 0.001 by one-way ANOVA followed by the Bonferroni-Dunn test *versus* no β-CD treatment.

To determine whether higher cholesterol levels lead to reduced exosome release, we carried out experiments to determine the effect of cholesterol depletion by treatment with β-cyclodextrin (β-CD) on exosome release, as demonstrated by flotillin/HSP90 release. Astrocytes were treated with β-CD at a final concentration of 0.1, 1, or 2 mM for 24 h at 37°C. The cellular cholesterol levels of astrocytes isolated from ApoE-KO and WT mice were significantly reduced in a β-CD-dose-dependent manner (**Figure 4F**). In both types of cultured astrocytes, exosome release increased when the cellular cholesterol level decreased, and the difference in exosome release between ApoE-deficient astrocytes and WT astrocytes reached similar levels with the treatment of β-CD at the concentrations of 0.1, 1, and 2 mM (**Figures 4A–C**). In contrast, the cellular levels of flotillin and HSP90 remained unchanged (**Figures 4A, D, E**). These results indicated that reduced cholesterol levels induce exosome release.

**Figure 5:**
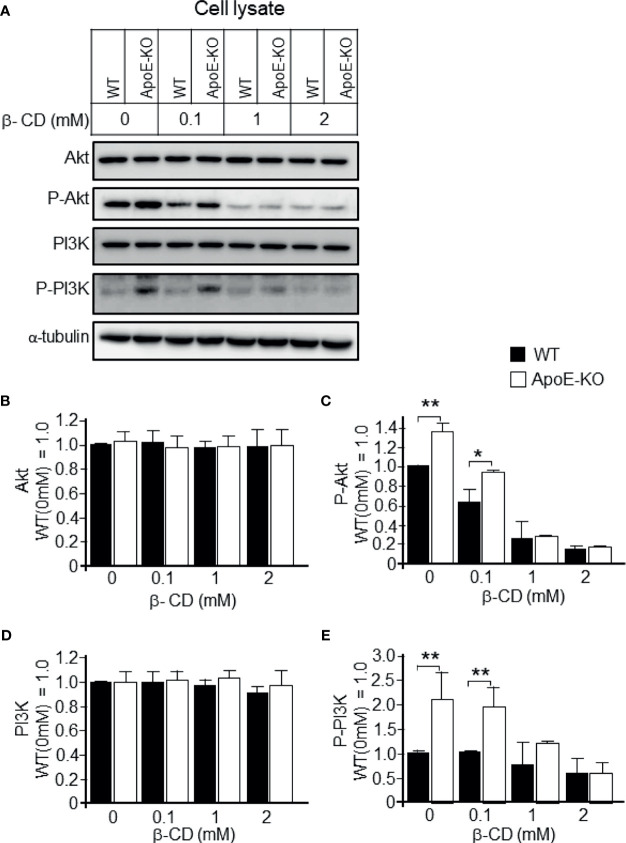
Effect of cholesterol depletion due to β-CD on phospho-PI3K and phospho-Akt. Astrocyte cultures prepared from WT and ApoE-KO mouse brain were treated with β-CD at the concentration of 0.1, 1, or 2 mM, and incubated for 24 h. Then, the CM and cell lysates were harvested and subjected to western blot analysis. **(A)** Western blot analysis was performed using antibodies against Akt, p-Akt, PI3K, p-PI3K, and α-tubulin as an internal control. **(B–E)** The intensity of each band was quantified by image analysis software (ImageJ 1.46r; Java 1.6.0-20 [64 bit]). Data are expressed as the mean ± SE. n = 3 each. *p < 0.05, **p < 0.01 by one-way ANOVA followed by the Bonferroni-Dunn test.

We further analyzed the effect of cholesterol depletion on cell signaling molecules. We observed that the astrocytes with lower levels of cholesterol had significantly reduced levels of the phosphorylated form of PI3K/Akt when compared to the control astrocytes without β-CD treatment (**Figures 5A, C, E**). However, there were no significant changes in the levels of total PI3K/Akt (pan-PI3K/Akt; **Figures 5B, D**).

**Figure 6:**
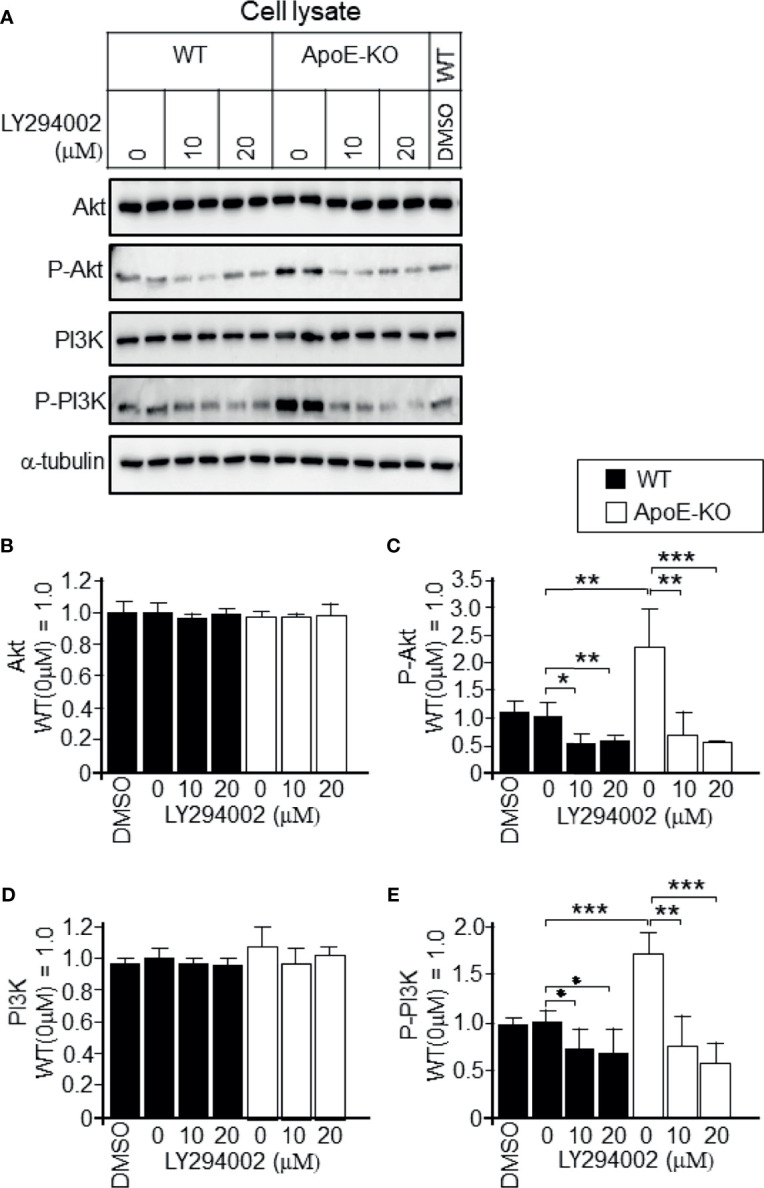
Attenuation of the activation of PI3K and Akt in ApoE-deficient astrocytes by a PI3K-specific inhibitor, LY294002. Astrocyte cultures prepared from WT and ApoE-KO mouse brain were treated with LY294002, a specific inhibitor of PI3K. The cultures were then incubated for another 2 days, and the cell lysates were harvested. **(A)** An equal amount of protein from each sample was subjected to western blot analysis using antibodies against Akt, p-Akt, PI3K, p-PI3K, and α-tubulin. **(B–E)** The intensity of each band was quantified by image analysis software (ImageJ 1.46r; Java 1.6.0-20 [64 bit]). Data are expressed as the mean ± SE. n = 3 each. *p < 0.05, **p < 0.01, ***p < 0.001 by one-way ANOVA followed by the Bonferroni-Dunn test.

**Figure 7:**
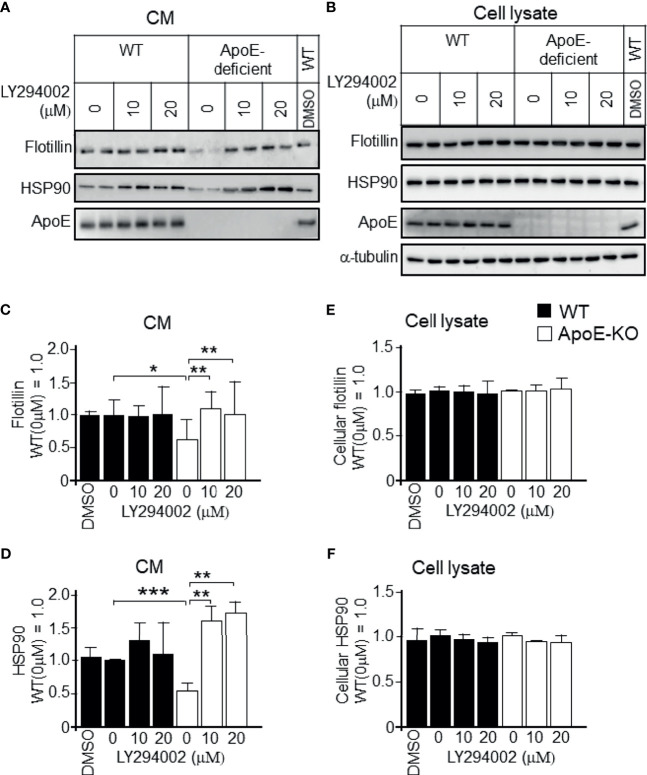
Recovery of the decreased exosome release by treatment with a PI3K-specific inhibitor, LY294002, in cultured ApoE-deficient astrocytes. Astrocyte cultures prepared from WT and ApoE-KO mouse brain were treated with LY294002, a specific inhibitor of PI3K. The cultures were then incubated for another 2 days, and the CM and cell lysates were harvested. **(A, B)** Western blot analysis of each sample was performed to determine the levels of flotillin, HSP90, and ApoE in the CM and cell lysates from the WT and ApoE-deficient astrocytes. **(C–F)** The intensity of each band was quantified by image analysis software (ImageJ 1.46r; Java 1.6.0-20 [64 bit]). Data are expressed as the mean ± SE. n = 3 each. *p < 0.05, **p < 0.01, ***p < 0.001 by one-way ANOVA followed by the Bonferroni-Dunn test.

To determine whether the increased levels of p-PI3K/p-Akt that were induced by higher levels of cellular cholesterol were responsible for the reduced exosome release, we treated astrocytes with PI3K-specific inhibitor LY294002 at the concentration of 10 or 20 µM. We confirmed that LY294002 treatment significantly reduced the p-PI3K and p-Akt levels in ApoE-deficient and WT astrocytes (**Figures 6A, C, E**); however, it did not have any effect on the total PI3K and Akt levels in either type of astrocytes (**Figures 6B, D**). We also examined whether the PI3K/Akt pathway is responsible for the regulation of exosome release. In the presence of a PI3K inhibitor, LY294002, the decreased levels of exosome release, as demonstrated by the flotillin and HSP90 release, in ApoE-deficient astrocytes, which have higher levels of cellular cholesterol, recovered without any changes in the cellular flotillin and HSP90 levels (**Figures 7A-F**). These results suggested that exosome release is regulated by cellular cholesterol *via* the PI3K/Akt signaling pathway.

**Figure 8:**
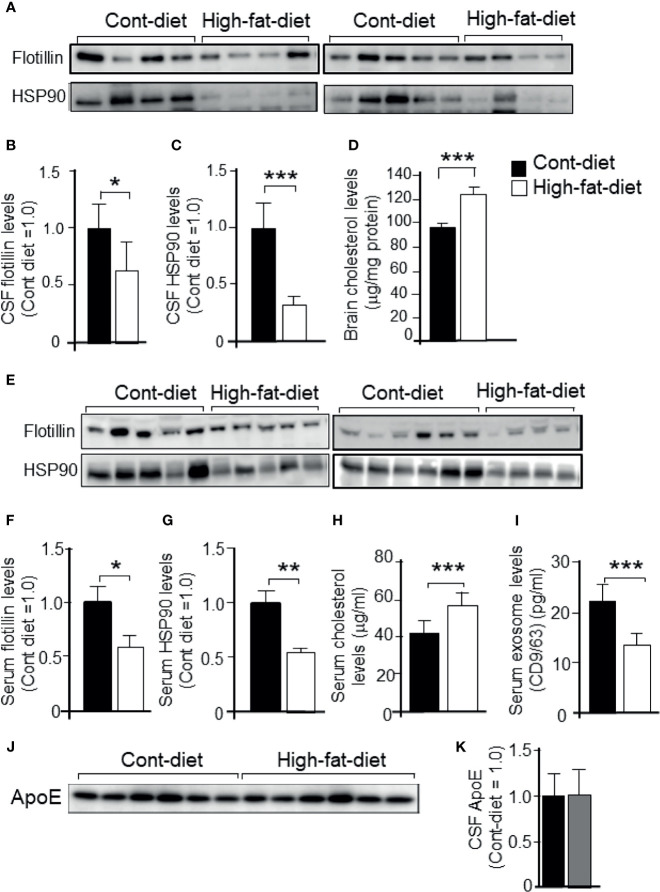
Exosome levels in the CSF and serum collected from mice that were fed a high-fat diet or control diet. Three-month-old WT mice were fed a high-fat diet or control diet for 4 months. At the age of 7 months, the mice were sacrificed, CSF was obtained from the cisterna magna, and serum was collected. Then, each sample was subjected to western blot analysis. **(A)** CSF samples were subjected to western blot analysis using antibodies against flotillin and HSP90. **(B, C)** The intensity of each band representing CSF flotillin and HSP90 was quantified by image analysis software (ImageJ 1.46r; Java 1.6.0-20 [64 bit]). **(D)** The cholesterol levels in mouse brain were determined by a cholesterol determination kit. **(E)** Serum samples were subjected to western blot analysis using antibodies against flotillin and HSP90. **(F, G)** The intensity of each band representing serum flotillin and HSP90 was quantified by image analysis software (ImageJ 1.46r; Java 1.6.0-20 [64 bit]). **(H)** The serum cholesterol concentrations were determined by a cholesterol determination kit. **(I)** Serum exosome levels were determined by a CD9/CD63 ELISA kit as described in the Experimental Procedures section. **(J, K)** ApoE levels in the CSF collected from mice fed with control diet and high-fat diet were determined by western blot analysis using anti-ApoE antibody. Data are expressed as the mean ± SE. *p < 0.05, **p < 0.01, ***p < 0.001 by Student’s *t-*test.

To investigate whether the cholesterol-dependent exosome release that was seen in cultures is also seen *in vivo*, 3-month-old WT mice were fed a high-fat diet or a control diet for 4 months. The basic formulation of a high-fat diet and a control diet are shown in **Tables 1**. At 7 months of age, the mice were sacrificed, and CSF from the cisterna magna, blood serum, and brain were collected. The samples were subjected to western blot analysis. We found that the levels of exosomes, as demonstrated by the flotillin and HSP90 levels, in CSF were significantly lower in the mice that were fed the high-fat diet than in those that were fed the control diet (**Figures 8A–C**). The cholesterol levels in brain were higher in the mice that were fed the high-fat diet than in those that were fed the control diet (**Figure 8D**). We also found that the serum levels of exosomes, as demonstrated by the flotillin and HSP90 levels, were significantly lower in the mice that were fed the high-fat diet than in those that were fed the control diet (**Figures 8E–G**). The levels of serum cholesterol were significantly higher in the mice that were fed the high-fat diet than in those that were fed the control diet (**Figure 8H**). We also analyzed the serum exosome levels using a CD9/CD63 exosome enzyme-linked immunosorbent assay (ELISA) kit. In accord with the results of the western blot analysis, the ELISA revealed that the serum exosome levels were significantly lower in the mice that were fed the high-fat diet than in those that were fed the control diet (**Figure 8I**). ApoE levels in the CSF collected from mice fed with control diet and high-fat diet were determined by western blot analysis using anti-ApoE antibody. The ApoE levels in the CSF of these mice show similar levels (**Figures 8J, K**).

**Table 1:** Basic formulation of high-fat diet and control diet.

Basic formulation	High-fat diet (HFD-60)	Control diet (AIN-93M)
	(g/kg diet)	(g/kg diet)
Milk Casein	256.0	140.0
L-Cystine	3.6	1.8
Maltodextrin	60.0	0.0
Cornstarch	0.0	465.692
α-Cornstarch	160.0	155.0
Sucrose	55.0	100.0
Soybean oil	20.0	4.0
Lard	330.0	0.0
Powdered Cellulose	66.1	50.0
mineral mix	35.0	35.0
Calcium carbonate	1.8	0.0
Vitamin mix	10.0	10.0
Choline bitartrate	2.5	2.5
tert-Butylhydroquinone	0.0	0.008
Total	1,000.0 g	1,000.0 g

## Discussion

In this study, we found that exosome release into the CM was significantly reduced in cultured astrocytes prepared from ApoE-KO mice when compared to those from WT mice. We also found that the decreased levels of exosome release in ApoE-deficient astrocytes were accompanied by increased levels of cellular cholesterol as well as phosphorylated PI3K and Akt. When the cellular cholesterol levels increased due to the treatment with cholesterol in astrocytes prepared from WT mice, the levels of exosome release decreased, while the levels of phosphorylated PI3K and Akt increased. The decreased levels of exosomes and the increased levels of cholesterol recovered to the control levels in ApoE-deficient astrocytes as a result of cholesterol depletion due to β-CD. Similarly, the decreased levels of exosomes and the increased levels of cholesterol recovered to the control levels in ApoE-deficient astrocytes as a result of treatment with the PI3K inhibitor, LY-294002. Moreover, we found that exosome release was decreased in the CSF and serum isolated from the WT mice that were fed the high-fat diet and had significantly increased cholesterol levels in the serum and brain. These lines of evidence suggest that increased levels of cholesterol in ApoE-deficient astrocytes stimulate the PI3K/Akt pathway, which in turn attenuates exosome release.

With respect to the increased levels of brain cholesterol in ApoE-deficient astrocytes, it may be possible that the cause was a lack of ApoE-mediated cholesterol efflux, as our previous study has shown (22). In support of this notion, it is known that ApoE is an important modulator of atherosclerosis as it reduces cholesterol accumulation in vessels, and spontaneous hyperlipidemia and atherosclerosis have been observed in mice lacking ApoE (30, 31).

The next question to be addressed was whether the attenuated exosome release and the accompanying enhancement of PI3K and Akt phosphorylation were caused by increased levels of cellular cholesterol, but not by the absence of ApoE. To answer this question, we treated cells with cholesterol and analyzed the levels of released exosomes, as well as PI3K and Akt phosphorylation. We found that the addition of cholesterol to astrocytes increased the levels of PI3K and Akt phosphorylation, and the cellular cholesterol levels significantly increased (**Figures 2** and **3**). In contrast, reducing the cellular cholesterol levels by β-CD treatment significantly enhanced exosome release and reduced the levels of phosphorylated PI3K/Akt (**Figures 4**, **5**). These results indicated that the increased levels of cholesterol, but not another function of ApoE, regulate the exosome release accompanied by enhanced phosphorylation of PI3K and Akt. As noted above, exosomes are rich in cholesterol, SM, glycosphingolipids, and phosphatidylserine, which are essential players in exosome biogenesis and release (7). Lipids, such as cholesterol, accumulate in intracellular vesicles under certain pathological conditions, such as Alzheimer’s disease and lysosomal storage disorders (32, 33). It has been reported that cholesterol regulates vesicular fusion with the plasma membrane, a step that is assumed to be critical for exosome release by controlling the content of specific proteins (34). However, no direct evidence showing the involvement of cholesterol in exosome release has yet been shown. Regarding lipid-mediated exosome release, there have been reports showing that exosome release is modulated in a sphingolipid-dependent manner (19), and that the activation of sphingosine 1-phosphate receptors mediates the maturation of exosomes (35). In addition to sphingolipid, in the present study, we showed for the first time the cellular cholesterol-dependent regulation of exosome release.

Next, we examined whether the cholesterol-dependent regulation of exosome release is mediated by PI3K/Akt activity using a specific inhibitor of PI3K, LY294002, in cultured WT and ApoE-deficient astrocytes. We found that the reduced levels of exosome release in ApoE-deficient astrocytes recovered to levels similar to those in WT astrocytes (**Figure 7C**). These results indicated that the PI3K/Akt pathway mediates the cholesterol-dependent reduction in the levels of exosome release from cultured astrocytes. Although the cholesterol-dependent regulation of PI3K/Akt activity has not been previously reported in neural cells, in agreement with our present results, previous studies in non-neuronal cells have shown that cholesterol activates PI3K/Akt signaling in human bone marrow mesenchymal stromal cells (36) and in human melanoma cells (37, 38). In contrast, cholesterol depletion in lipid rafts of cell membranes due to methylated β-CD treatment induces apoptosis through the PI3K-Akt pathway in non-neuronal cell lines (39). Although the mechanisms underlying exosome formation and release are poorly understood, it is believed that several pathways and molecules are involved in exosome biogenesis and release (40). Our findings suggest that involvement of the PI3K/Akt pathway is one of them.

Taken together, we showed that the level of cellular cholesterol regulates exosome release by modulating the PI3K/Akt pathway. The role and function of exosomes in brain are not well understood; however, several reports have showed that flotillin plays a role in axonal regeneration (41, 42). There are also studies reporting the involvement of flotillin in neurodegenerative diseases such as Alzheimer's disease (43) and Parkinson's disease (44). Although the roles of exosomes in diseases and the maintenance of cellular functions are not well understood, our findings from the present study provide new insights into the molecular mechanisms that regulate exosome release from cells.

## Conclusion

In summary, we found that cellular cholesterol regulates exosome release from astrocytes in culture; that is higher levels of cholesterol attenuates exosome release and lower levels of cholesterol enhances exosome release. These cholesterol-dependent regulation of exosome release is mediated by PI3K/Akt pathways. The cholesterol-dependent regulation of exosome release in the CSF and serum was also confirmed by *in vivo* experiments. These results suggest that exosome release is regulated by cellular cholesterol *via* stimulation of the PI3K/Akt signal pathway.

## Data Availability Statement

The original contributions presented in the study are included in the article/supplementary material. Further inquiries can be directed to the corresponding author.

## Ethics Statement

The animal study was reviewed and approved by Nagoya City University Animal Ethics Committee.

## Author Contributions

MM designed research. MA, TN, TF, YG, YC and KZ performed research. MA, TN, KZ and MM analyzed data. MA, TN and MM wrote the paper. All authors contributed to the article and approved the submitted version.

## Funding

This work was supported by Grant-in-Aid for Scientific Research B (16H05559) (to MM) and for Challenging Exploratory Research (15K15712) (to MM) from the Ministry of Education, Culture, Sports, Science and Technology, Japan. This study was also supported by AMED “Project of Translational and Clinical Research Seed A” under grant number A-128, and “Research and Development Grants for Dementia” under grant number 21dk0207050h0002 (to MM).

## Conflict of Interest

The authors declare that the research was conducted in the absence of any commercial or financial relationships that could be construed as a potential conflict of interest.

## Publisher’s Note

All claims expressed in this article are solely those of the authors and do not necessarily represent those of their affiliated organizations, or those of the publisher, the editors and the reviewers. Any product that may be evaluated in this article, or claim that may be made by its manufacturer, is not guaranteed or endorsed by the publisher.

## References

[1] Yanez-Mo M Siljander PR Andreu Z Zavec AB Borras FE Buzas EI. Biological Properties of Extracellular Vesicles and Their Physiological Functions. J Extracell Vesicles (2015) 4:27066. doi: 10.3402/jev.v4.27066 25979354 PMC4433489

[2] van Niel G D’Angelo G Raposo G. Shedding Light on the Cell Biology of Extracellular Vesicles. Nat Rev Mol Cell Biol (2018) 19(4):213–28. doi: 10.1038/nrm.2017.125 29339798

[3] Thery C Ostrowski M Segura E. Membrane Vesicles as Conveyors of Immune Responses. Nat Rev Immunol (2009) 9(8):581–93. doi: 10.1038/nri2567 19498381

[4] Pisitkun T Shen RF Knepper MA. Identification and Proteomic Profiling of Exosomes in Human Urine. Proc Natl Acad Sci U.S.A. (2004) 101(36):13368–73. doi: 10.1073/pnas.0403453101 PMC516573 15326289

[5] Laulagnier K Motta C Hamdi S Roy S Fauvelle F Pageaux JF. Mast Cell- and Dendritic Cell-Derived Exosomes Display a Specific Lipid Composition and an Unusual Membrane Organization. Biochem J (2004) 380(Pt 1):161–71. doi: 10.1042/BJ20031594 PMC1224152 14965343

[6] de Gassart A Geminard C Fevrier B Raposo G Vidal M. Lipid Raft-Associated Protein Sorting in Exosomes. Blood (2003) 102(13):4336–44. doi: 10.1182/blood-2003-03-0871 12881314

[7] Skotland T Hessvik NP Sandvig K Llorente A. Exosomal Lipid Composition and the Role of Ether Lipids and Phosphoinositides in Exosome Biology. J Lipid Res (2019) 60(1):9–18. doi: 10.1194/jlr.R084343 30076207 PMC6314266

[8] Ratajczak J Miekus K Kucia M Zhang J Reca R Dvorak P. Embryonic Stem Cell-Derived Microvesicles Reprogram Hematopoietic Progenitors: Evidence for Horizontal Transfer of mRNA and Protein Delivery. Leukemia (2006) 20(5):847–56. doi: 10.1038/sj.leu.2404132 16453000

[9] Valadi H Ekstrom K Bossios A Sjostrand M Lee JJ Lotvall JO. Exosome-Mediated Transfer of mRNAs and microRNAs is a Novel Mechanism of Genetic Exchange Between Cells. Nat Cell Biol (2007) 9(6):654–9. doi: 10.1038/ncb1596 17486113

[10] Skog J Wurdinger T van Rijn S Meijer DH Gainche L Sena-Esteves M. Glioblastoma Microvesicles Transport RNA and Proteins That Promote Tumour Growth and Provide Diagnostic Biomarkers. Nat Cell Biol (2008) 10(12):1470–6. doi: 10.1038/ncb1800 PMC3423894 19011622

[11] Raposo G Nijman HW Stoorvogel W Liejendekker R Harding CV Melief CJ. B Lymphocytes Secrete Antigen-Presenting Vesicles. J Exp Med (1996) 183(3):1161–72. doi: 10.1084/jem.183.3.1161 PMC2192324 8642258

[12] Abdullah M Kimura N Akatsu H Hashizume Y Ferdous T Tachita T. Flotillin is a Novel Diagnostic Blood Marker of Alzheimer’s Disease. J Alzheimers Dis (2019) 72(4):1165–76. doi: 10.3233/JAD-190908 31683489

[13] Admyre C Johansson SM Qazi KR Filen JJ Lahesmaa R Norman M. Exosomes With Immune Modulatory Features are Present in Human Breast Milk. J Immunol (2007) 179(3):1969–78. doi: 10.4049/jimmunol.179.3.1969 17641064

[14] Abdullah M Takase H Nunome M Enomoto H Ito J Gong JS. Amyloid-Beta Reduces Exosome Release From Astrocytes by Enhancing JNK Phosphorylation. J Alzheimers Dis (2016) 53(4):1433–41. doi: 10.3233/JAD-160292 27392863

[15] Yuana Y Sturk A Nieuwland R. Extracellular Vesicles in Physiological and Pathological Conditions. Blood Rev (2013) 27(1):31–9. doi: 10.1016/j.blre.2012.12.002 23261067

[16] Tamai K Tanaka N Nakano T Kakazu E Kondo Y Inoue J. Exosome Secretion of Dendritic Cells is Regulated by Hrs, an ESCRT-0 Protein. Biochem Biophys Res Commun (2010) 399(3):384–90. doi: 10.1016/j.bbrc.2010.07.083 20673754

[17] Colombo M Moita C van Niel G Kowal J Vigneron J Benaroch P. Analysis of ESCRT Functions in Exosome Biogenesis, Composition and Secretion Highlights the Heterogeneity of Extracellular Vesicles. J Cell Sci (2013) 126(Pt 24):5553–65. doi: 10.1242/jcs.128868 24105262

[18] Subra C Grand D Laulagnier K Stella A Lambeau G Paillasse M. Exosomes Account for Vesicle-Mediated Transcellular Transport of Activatable Phospholipases and Prostaglandins. J Lipid Res (2010) 51(8):2105–20. doi: 10.1194/jlr.M003657 PMC2903822 20424270

[19] Yuyama K Sun H Mitsutake S Igarashi Y. Sphingolipid-Modulated Exosome Secretion Promotes Clearance of Amyloid-Beta by Microglia. J Biol Chem (2012) 287(14):10977–89. doi: 10.1074/jbc.M111.324616 PMC3322859 22303002

[20] Yuyama K Sun H Mikami D Mioka T Mukai K Igarashi Y. Lysosomal-Associated Transmembrane Protein 4B Regulates Ceramide-Induced Exosome Release. FASEB J (2020) 34(12):16022–33. doi: 10.1096/fj.202001599R 33090522

[21] Hatters DM Peters-Libeu CA Weisgraber KH. Apolipoprotein E Structure: Insights Into Function. Trends Biochem Sci (2006) 31(8):445–54. doi: 10.1016/j.tibs.2006.06.008 16820298

[22] Michikawa M Fan QW Isobe I Yanagisawa K. Apolipoprotein E Exhibits Isoform-Specific Promotion of Lipid Efflux From Astrocytes and Neurons in Culture. J Neurochem (2000) 74(3):1008–16. doi: 10.1046/j.1471-4159.2000.0741008.x 10693931

[23] Pitas RE Boyles JK Lee SH Hui D Weisgraber KH. Lipoproteins and Their Receptors in the Central Nervous System. Characterization of the Lipoproteins in Cerebrospinal Fluid and Identification of Apolipoprotein B,E(LDL) Receptors in the Brain. J Biol Chem (1987) 262(29):14352–60. doi: 10.1016/S0021-9258(18)47945-8 3115992

[24] Krimbou L Denis M Haidar B Carrier M Marcil M Genest J Jr. Molecular Interactions Between apoE and ABCA1: Impact on apoE Lipidation. J Lipid Res (2004) 45(5):839–48. doi: 10.1194/jlr.M300418-JLR200 14754908

[25] Ito J Zhang LY Asai M Yokoyama S. Differential Generation of High-Density Lipoprotein by Endogenous and Exogenous Apolipoproteins in Cultured Fetal Rat Astrocytes. J Neurochem (1999) 72(6):2362–9. doi: 10.1046/j.1471-4159.1999.0722362.x 10349845

[26] Gong JS Kobayashi M Hayashi H Zou K Sawamura N Fujita SC. Apolipoprotein E (ApoE) Isoform-Dependent Lipid Release From Astrocytes Prepared From Human ApoE3 and ApoE4 Knock-in Mice. J Biol Chem (2002) 277(33):29919–26. doi: 10.1074/jbc.M203934200 12042316

[27] Minagawa H Watanabe A Akatsu H Adachi K Ohtsuka C Terayama Y. Homocysteine, Another Risk Factor for Alzheimer Disease, Impairs Apolipoprotein E3 Function. J Biol Chem (2010) 285(49):38382–8. doi: 10.1074/jbc.M110.146258 PMC2992271 20889503

[28] Ju R Zhuang ZW Zhang J Lanahan AA Kyriakides T Sessa WC. Angiopoietin-2 Secretion by Endothelial Cell Exosomes: Regulation by the Phosphatidylinositol 3-Kinase (PI3K)/Akt/endothelial Nitric Oxide Synthase (eNOS) and Syndecan-4/Syntenin Pathways. J Biol Chem (2014) 289(1):510–9. doi: 10.1074/jbc.M113.506899 PMC3879572 24235146

[29] Mollinedo F Gajate C. Lipid Rafts as Signaling Hubs in Cancer Cell Survival/Death and Invasion: Implications in Tumor Progression and Therapy: Thematic Review Series: Biology of Lipid Rafts. J Lipid Res (2020) 61(5):611–35. doi: 10.1194/jlr.TR119000439 PMC7193951 33715811

[30] Zhang SH Reddick RL Piedrahita JA Maeda N. Spontaneous Hypercholesterolemia and Arterial Lesions in Mice Lacking Apolipoprotein E. Science (1992) 258(5081):468–71. doi: 10.1126/science.1411543 1411543

[31] Gaudreault N Kumar N Posada JM Stephens KB Reyes de Mochel NS Eberle D. ApoE Suppresses Atherosclerosis by Reducing Lipid Accumulation in Circulating Monocytes and the Expression of Inflammatory Molecules on Monocytes and Vascular Endothelium. Arterioscler Thromb Vasc Biol (2012) 32(2):264–72. doi: 10.1161/ATVBAHA.111.238964 PMC3275345 22053073

[32] Distl R Meske V Ohm TG. Tangle-Bearing Neurons Contain More Free Cholesterol Than Adjacent Tangle-Free Neurons. Acta Neuropathol (2001) 101(6):547–54. doi: 10.1007/s004010000314 11515782

[33] Kuech EM Brogden G Naim HY. Alterations in Membrane Trafficking and Pathophysiological Implications in Lysosomal Storage Disorders. Biochimie (2016) 130:152–62. doi: 10.1016/j.biochi.2016.09.011 27664461

[34] Ammar MR Kassas N Chasserot-Golaz S Bader MF Vitale N. Lipids in Regulated Exocytosis: What are They Doing? Front Endocrinol (Lausanne) (2013) 4:125. doi: 10.3389/fendo.2013.00125 24062727 PMC3775428

[35] Kajimoto T Okada T Miya S Zhang L Nakamura S. Ongoing Activation of Sphingosine 1-Phosphate Receptors Mediates Maturation of Exosomal Multivesicular Endosomes. Nat Commun (2013) 4:2712. doi: 10.1038/ncomms3712 24231649

[36] Baker N Sohn J Tuan RS. Promotion of Human Mesenchymal Stem Cell Osteogenesis by PI3-Kinase/Akt Signaling, and the Influence of Caveolin-1/Cholesterol Homeostasis. Stem Cell Res Ther (2015) 6:238. doi: 10.1186/s13287-015-0225-8 26626726 PMC4667507

[37] Yamauchi Y Furukawa K Hamamura K. Positive Feedback Loop Between PI3K-Akt-Mtorc1 Signaling and the Lipogenic Pathway Boosts Akt Signaling: Induction of the Lipogenic Pathway by a Melanoma Antigen. Cancer Res (2011) 71(14):4989–97. doi: 10.1158/0008-5472.CAN-10-4108 21632551

[38] Xu J Qian J Xie X Lin L Ma J Huang Z. High Density Lipoprotein Cholesterol Promotes the Proliferation of Bone-Derived Mesenchymal Stem Cells *via* Binding Scavenger Receptor-B Type I and Activation of PI3K/Akt, MAPK/ERK1/2 Pathways. Mol Cell Biochem (2012) 371(1-2):55–64. doi: 10.1007/s11010-012-1422-8 22886428

[39] Motoyama K Kameyama K Onodera R Araki N Hirayama F Uekama K. Involvement of PI3K-Akt-Bad Pathway in Apoptosis Induced by 2,6-Di-O-Methyl-Beta-Cyclodextrin, Not 2,6-Di-O-Methyl-Alpha-Cyclodextrin, Through Cholesterol Depletion From Lipid Rafts on Plasma Membranes in Cells. Eur J Pharm Sci (2009) 38(3):249–61. doi: 10.1016/j.ejps.2009.07.010 19664706

[40] Hessvik NP Llorente A. Current Knowledge on Exosome Biogenesis and Release. Cell Mol Life Sci (2018) 75(2):193–208. doi: 10.1007/s00018-017-2595-9 28733901 PMC5756260

[41] Schulte T Paschke KA Laessing U Lottspeich F Stuermer CA. Reggie-1 and Reggie-2, Two Cell Surface Proteins Expressed by Retinal Ganglion Cells during Axon Regeneration. Development (1997) 124(2):577–87. doi: 10.1242/dev.124.2.577 9053333

[42] Stuermer CA. How Reggies Regulate Regeneration and Axon Growth. Cell Tissue Res (2012) 349(1):71–7. doi: 10.1007/s00441-012-1343-6 22350847

[43] Girardot N Allinquant B Langui D Laquerriere A Dubois B Hauw JJ. Accumulation of Flotillin-1 in Tangle-Bearing Neurones of Alzheimer's Disease. Neuropathol Appl Neurobiol (2003) 29(5):451–61. doi: 10.1046/j.1365-2990.2003.00479.x 14507337

[44] Jacobowitz DM Kallarakal AT. Flotillin-1 in the Substantia Nigra of the Parkinson Brain and a Predominant Localization in Catecholaminergic Nerves in the Rat Brain. Neurotox Res (2004) 6(4):245–57. doi: 10.1007/BF03033435 15545008

